# Mutational Profiling of Kinases in Human Tumours of Pancreatic Origin Identifies Candidate Cancer Genes in Ductal and Ampulla of Vater Carcinomas

**DOI:** 10.1371/journal.pone.0012653

**Published:** 2010-09-08

**Authors:** Vincenzo Corbo, Rossana Ritelli, Stefano Barbi, Niccola Funel, Daniela Campani, Alberto Bardelli, Aldo Scarpa

**Affiliations:** 1 ARC-NET Research Center, University of Verona, Policlinico G.B. Rossi, Verona, Italy; 2 Department of Pathology, University of Verona, Policlinico G.B. Rossi, Verona, Italy; 3 Department of Pathology, University of Pisa, Pisa, Italy; 4 Laboratory of Molecular Genetics, Institute for Cancer Research and Treatment, University of Torino Medical School, Candiolo, Italy; Technische Universität München, Germany

## Abstract

**Background:**

Protein kinases are key regulators of cellular processes (such as proliferation, apoptosis and invasion) that are often deregulated in human cancers. Accordingly, kinase genes have been the first to be systematically analyzed in human tumors leading to the discovery that many oncogenes correspond to mutated kinases. In most cases the genetic alterations translate in constitutively active kinase proteins, which are amenable of therapeutic targeting. Tumours of the pancreas are aggressive neoplasms for which no effective therapeutic strategy is currently available.

**Methodology/Principal Findings:**

We conducted a DNA-sequence analysis of a selected set of 35 kinase genes in a panel of 52 pancreatic exocrine neoplasms, including 36 pancreatic ductal adenocarcinoma, and 16 ampulla of Vater cancer. Among other changes we found somatic mutations in *ATM*, *EGFR, EPHA3, EPHB2*, and *KIT*, none of which was previously described in cancers.

**Conclusions/Significance:**

Although the alterations identified require further experimental evaluation, the localization within defined protein domains indicates functional relevance for most of them. Some of the mutated genes, including the tyrosine kinases *EPHA3* and *EPHB2*, are clearly amenable to pharmacological intervention and could represent novel therapeutic targets for these incurable cancers.

## Introduction

Protein kinases are key regulators of different and complex cellular processes such as cell cycle progression, differentiation, apoptosis and invasion [1]–[4]. The protein kinase complement (defined as “kinome”) represents a significant fraction of the human genome, and recently Manning *et al*. organized it into a dendogram containing nine broad groups of genes [5]. Alterations in a kinase gene can lead to an aberrant protein activity, which may have a role in cancer initiation and progression [6], [7]. These alterations, including point mutations and deletions in conserved domains, often result in constitutively activated kinases, which are potential therapeutic targets for cancer treatment. Indeed, there are several small molecule inhibitors currently in use or being evaluated in clinical trials [8]–[10]. Mutations in a kinase gene may also result in its inactivation, as for genes involved in the maintenance of genome stability [11], [12] or controlling cell-cell communication [13]. In recent years, extensive sequence analysis of tumour genomes and particularly of kinase gene family has been conducted in different epithelial tumours leading to the identification of different somatic mutations [14]–[22]. These works point to a subset of kinase genes with known or potential relationship with solid tumour development, as they display a relatively high frequency of mutations.

To determine the presence of mutations potentially relevant as therapeutic targets, we conducted a DNA-sequence analysis of a selected set of kinase genes in a panel of two different pancreatic neoplasms, including pancreatic ductal adenocarcinoma (PDAC), and ampulla of Vater cancer (AVC). For these tumour types no effective therapeutic agents are currently available [23], [24]. For example, PDAC is highly aggressive and resistant to conventional and targeted therapeutic agents, resulting in a dismal 5-year survival rate of 3% to 5% [24]. Here we present the mutational profile of 35 genes belonging to the kinase gene families in PDAC and AVC. Specifically we found non-synonymous mutations in the following genes: *ATM, BRAF, EGFR, EPHA3, EPHB2, ERBB2, FGFR2, KIT*, and *PIK3CA*. Among these changes, there were both well-characterized mutations and mutations affecting aminoacid not previously found to be mutated in human cancers.

## Materials and Methods

### Ethics Statement

All research involving human participants was approved by the University and Hospital Trust's Institutional Board. Informed consent was obtained in writing from living patients or relatives for all tissues used in this study.

### Samples

The panel of 52 pancreatic cancer samples was obtained from the tumour bank maintained by the Department of Pathology, Section of Anatomic Pathology, University of Verona (Verona, Italy), except for six of pancreatic adenocarcinoma samples provided by Dr. Daniela Campani (University of Pisa). Samples were collected according to the ethical requirements and regulations of the review boards of both the University of Verona (Verona, Italy) and the University of Pisa (Pisa, Italy). This panel included 36 PDAC and 16 AVC (see supplementary **Table S1** for detailed clinical information of cancer samples). The 23 PDAC tumors were passaged in vitro as cell lines or in nude mice as xenografts to remove contaminating non-neoplastic cells [25]. Cell lines were harvested after a maximum of six *in vitro* passages for nucleic acid preparation. Thirteen PDAC samples were previously described human pancreatic tumour cell lines [26] (see supplementary **Table S2** for details). Tissue samples estimated to contain more than 80% of tumour cells were used. Genomic DNA was isolated using Dneasy Blood and Tissue kit (Qiagen, Milan, Italy). Matched normal DNA was used to determine whether the mutations identified were somatic or germline. No matched normal sample was available for the 13 established cell lines included in the study. Genomic DNA was further isolated after cryostat enrichment from frozen tissues of primary PDAC to confirm the mutations eventually identified in the corresponding xenografted tumors.

### Genes, PCR and Sequencing

Thirty-five genes belonging to the protein kinase gene family were chosen on the basis of their high frequency of mutations in human cancers other than pancreatic as assessed in previous works [14]–[22]. These genes were: *AKT2, ATM, ATR, AURKC, BRAF, BRD2, DDR1, DYRK2, EGFR, EPHA3, EPHA5, EPHB6, EPHB2, ERBB2, ERBB4, FGFR1, FGFR2, FGFR3, FGFR4, FLT1, FLT3, FRAP1, KDR, KIT, MAP2K4, MET, NTRK2, NTRK3, PAK4, PDGFRA, PDPK1, PI3KCA, RPS6KC1, STK11, TGFBR2*. Primers for amplification and sequencing of DNA were designed using the Primer3 program (http://frodo.wi.mit.edu/cgi-bin/primer3/primer3_www.cgi) and refer to National Center for Biotechnology Information (NCBI, http://www.ncbi.nlm.nih.gov) reference sequence files with the Gene and Transcript ID (RefSeq) provided in supplementary **Table S3**. PCR primers were designed to amplify the selected exons and the flanking intronic sequences, including splicing donor and acceptor regions. PCR products were ∼400 bp in length, with multiple overlapping amplimers for larger exons. PCR conditions, purification, and direct sequencing have been previously described [14].

### Data analysis

Sequence differences to the NCBI reference sequence were identified via manual inspection of aligned electropherograms assisted by the Mutation Surveyor software package (SoftGenetics, State College, PA). The genetic alteration identified were cross-referenced to variant information from the NCBI SNP database, the Ensemble Genome Browser (http://www.ensembl.org), The Swiss-Prot (http://ca.expasy.org) and GeneBank databases http://www.ncbi.nlm.nih.gov/Genbank), the COSMIC database (http://www.sanger.ac.uk/genetics/CGP/cosmic), and literature. In addition to non-synonymous genetic alterations, we detected numerous silent sequence variations that were analyzed using the ASSP (http://www.es.embnet.org/~mwang/assp.html) sequence analysis tool to exclude that cryptic splices sites may be activated. These silent mutations are not presented and further analyzed here. All new sequence data has been deposited in GenBank.

## Results and Discussion

We performed a mutational profiling of 35 kinase genes in a panel of 36 PDAC, and 16 AVC, including primary tumours, xenografts and cell lines. For each gene, all exons in which somatic mutation had been previously identified were analyzed. Exon specific primers were designed to amplify and sequence the coding region, and at least 15 intronic bases at both the 5′ and 3′ ends, including the splicing donor and acceptor sites. A total of 8,321 PCR products, spanning over 3 Mb of tumour genomic DNA, were generated and subjected to direct sequencing. Of the 147 exons extracted, 92% furnished good sequence traces (i.e., more than 90% of bases in the target region had a Phred score (defined as −10[log_10_(raw per-base error)]) of at least 20 in at least three-quarters of the samples analyzed), and therefore were analyzed searching for specific mutations. Changes previously described as single nucleotide polymorphisms (SNPs) were excluded from further analysis. To ensure that the eventually observed mutations were not PCR or sequencing artifacts, amplicons were independently re-amplified and re-sequenced in the corresponding tumours. All verified changes were resequenced in parallel with the matched normal DNA, to distinguish between somatic mutations and SNPs not previously described.

This approach led to the identification of a total of 10 different non-synonymous mutations (**Table 1**). Among these mutations, 9 were missense; one was a small insertion, whilst no mutations were found in the splice sites or UTR regions (Table 1).

**Table 1:** Mutations Indentified in Protein Kinase Genes.

Gene	Nucleotide Change^a^	Amino acid change^a^	Mutation Type	Zygosity^c^	Tumor Type^d^	Sample
*ATM*	c.2879 C>T	p.R823C	Missense	Heterozygous	AVC	107p
*BRAF*	c.1452 G>T	p.G464V	Missense	Homozygous	PDAC	377
*EGFR*	c.2689 C>T	p.L815F	Missense	Heterozygous	PDAC	369
*EGFR*	g.173783-173784insA	892fs896stop^b^	Insertion	Heterozygous	AVC	160p
*EPHA3*	c.846 G>T	p.K207N	Missense	Heterozygous	PDAC	549
*EPHA3*	c.846 G>T	p.K207N	Missense	Heterozygous	AVC	135p
*EPHB2*	c.865 G>C	p.D283H	Missense	Heterozygous	PDAC	549
*EPHB2*	c.865 G>C	p.D283H	Missense	Heterozygous	PDAC	370
*ERBB2*	c.2759 G>T	p.V777L	Missense	Heterozygous	AVC	119p
*FGFR2*	c.2337 C>T	p.P582L	Missense	n.d.	PDAC	PT45
*KIT*	c.2240 G>A	p.R740K	Missense	Heterozygous	PDAC	PP161
*PI3KCA*	c.3297 A>G	p.H1047R	Missense	n.d.	PDAC	GER

NOTE: The mutations are listed by gene alongside the samples in which they were found. The nucleotide numbering uses the A of the ATG translation initiation start site as nucleotide + 1, based on reference sequences provided in Supplementary Table S3.

a c., cDNA sequence; g., genomic sequence; ins, insertion; p., protein sequence; fs, frameshift mutation.

b The insertion of the nucleotide A causes a frameshift and leads to a premature stop codon at amino acid 896.

c n.d., not determined.

d AVC, ampulla of Vater cancer; PDAC, pancreatic ductal adenocarcinoma.

e The genetic alteration identified were cross-referenced with variant information from databases and literature (see Materials and Methods section for details).

**Figure 1:**
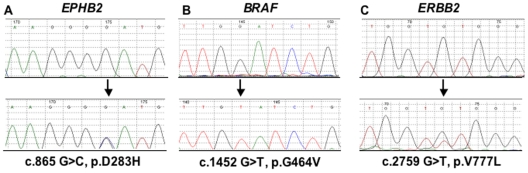
Examples of somatic mutations in *EPHB2*, *BRAF*, and *ERBB2*. *Bottom*, chromatogram of the sequence of a tumor sample; *top*, chromatogram of the matched normal. Arrows indicate the location of missense mutation. Number above the sequence traces are part of the software output. The nucleotide numbering uses the A of the ATG translation initiation start site as nucleotide +1, based on reference sequences provided in Supplementary Table S3. **A**, mutation in PDAC; **B**, mutation in PDAC; **C**, mutation in AVC. *Abbreviations:* g., genomic sequence; p., protein sequence.

With regard to PDAC we analyzed a total of 36 samples, 13 of which were established cell lines. A total of 7 different missense mutations affecting the following genes were found: *BRAF, EGFR, EPHA3, EPHB2, FGFR2, KIT, PIK3CA* (Table 1). Of these mutations *FGFR2^P582L^* and *PIK3CA* ^H1047R^ were identified in adenocarcinoma cell lines (PT45 and GER, respectively) for which no matched normal sample was available. Therefore, the somatic status of these mutations could not be ascertained. The *PIK3CA* ^H1047R^ mutation has been previously related to cancer and extensively characterized [27]–[30]. Although mutations of *FGFR2* have been previously found in human cancers [16], [31], [32], no mutations of this gene have been reported to be associated with pancreatic cancers to date. Our characterization of established pancreatic tumour cell lines with the respect to the genetic alterations in this potential cancer target family finally provides suitable cell systems for data interpretation, target validation, as well as preclinical models for the development of novel targeted cancer drugs. *BRAF* ^G464V^ has been previously related to cancer and characterized [33]. The finding of a *BRAF* mutation in PDAC is somehow expected since its related pathway is altered in almost all the pancreatic adenocarcinomas [34], although individual *BRAF* mutations are quite infrequent in this type of cancer and generally occur in tumours that lack *KRAS* mutations [35], [36]. Interestingly, the mutation we found was in homozygous state (**Figure 1B**), which is not expected for a protein that acts in a dominant fashion. The same missense somatic mutations of *EPHB2* (D283H) were found in two different PDAC samples (**Figure 1A**), one of which also displayed a missense mutation in *EPHA3* (K207N). The mutations identified in *BRAF* and *EPHB2* were re-analyzed in the corresponding primary tumours (Supplementary **Figure S1**) to further confirm the presence of mutations and thus exclude the possibility that the genetic alterations could be the consequence of the graft in nude mice. In particular, the sequencing analysis of the primary cancer corresponding to xenograft 377 (Table 1) also confirmed the homozygous state of the mutation identified in the *BRAF* gene. The Eph receptors represent the largest subfamily of trans-membrane tyrosine kinase receptors and are primary involved in the process of cell adhesion and migration during development, homeostasis and disease [13], [37]. In this study, we report the mutational profile of four members of this family (*EPHA3*, *EPHA7*, *EPHB2*, and *EPHB6*), which have been found frequently mutated or silenced in previous study on human cancers [13]. For example, mutations of EPHB2 that presumably lead to a loss of activity have been found in colorectal and prostate cancer [38], [39], whilst mutations in *EPHA3* have been described in melanoma, lung and colorectal cancer [14]–[16], [19]. Thus far, no mutations of *EPHB2* have been reported to be associated with pancreatic cancer. Otherwise, a very recent work that deeply analyzed the protein-coding genes in pancreatic adenocarcinomas reported the occurrence of an intronic point mutation of *EPHA3* [34]. The mutations of *EPHB2* and *EPHA3* we found in this study localized in the evolutionary conserved Cys-rich extracellular domain that is thought to be involved in determining the binding affinity to their ligands as well as the tetramerization of activated receptors [40]. Indeed, these aminoacid changes (D283H and K207N) may affect the binding of the receptors to their ligands and therefore possibly disrupt the normal signalling cascade. Furthermore, increasing evidence suggest a role of ephrin receptor/ephrin system in invasiveness in cancer as well as its potential relevance for therapeutic targeting [41]. Mutations of EGFR (L815F) affecting the kinase domain was harboured in only one of the PDAC samples according to the low incidence of EGFR somatic alterations found in pancreatic cancer by others [42], [43]. Finally, we report for the first time a somatic mutation in the kinase domain of KIT (R740K) in PDAC cancer. *KIT*, also designated as *CD117*, is frequently affected by activating mutations in gastro-intestinal stromal tumours [44], [45], thus representing a logical therapeutic target for this malignancy [46]. Although several studies suggest an involvement of KIT in pancreatic carcinogenesis, no somatic mutations have been previously found [47]–[49].

Concerning AVC we analyzed a total of 16 samples, including 15 primary tumours and one cell line. We found a total of four different somatic mutations, of which three were missense and one was a small insertion, affecting the following genes (Table 1): *ATM, EGFR, EPHA3*, and *ERBB2*. The somatic mutation *ERBB2* ^V777L^ has been previously found in human cancers [50], [51]. Furthermore, a careful analysis of *ERBB2* sequence electropherogram showed that the peak corresponding to the mutation was minor as compared to its wild type counterpart (**Figure 1C**). This suggest the occurrence of this variant only in a subpopulation of tumour cells of the samples. One of the most interesting alterations we found was the insertion occurring in the exon 22 of EGFR that leads to a premature stop codon at aminoacid 896 within the catalytic domain of the protein. This mutation however needs further evaluation to determine its functional significance. Interestingly, the *EPHA3* ^K207N^ mutation has also been detected in a PDAC sample. This possibly suggests a partial overlapping spectrum of genetic alteration underlying these different pancreatic cancer subtypes unless these are mutations occurring by chance.

In conclusion, in this study we identified 10 different mutations affecting 9 kinase genes in pancreatic ductal adenocarcinoma and ampulla of Vater cancers. No definite pattern of somatic mutations was identified for each tumour types and only one alteration (*EPHA3* ^K207N^) showed overlap between the tumor types analyzed. In agreement with the results from previous studies we observed a low frequency of specific somatic mutations in kinase genes [15], [16], [18]–[22], [34]. Except for the *FGFR2* and *PIK3CA* mutations that affected human tumour cell lines, the remaining 8 mutations were found in samples derived from primary tumours and were somatic in origin as assessed by the sequencing of the matched normal DNA (data not shown). With regard to PDAC recently Jones *et al.* performed a comprehensive genetic analysis leading to the identification of a defined set of partially overlapping signalling pathways that were altered, despite the fact that the alterations affecting the individual component varied widely between individual tumours [34]. Indeed, none of the somatic mutations described by Jones *et al* were found in our analyses and viceversa. Our and previous results thus suggest that a deep genetic analysis for each gene could be performed in a large series of pancreatic ductal adenocarcinoma samples to unravel the contribution of a specific gene to pancreatic tumorigenesis.

Finally, none of the alterations we found in primary tumours were previously described in cancers. These alterations require further experimental evaluation to determine their functional relevance and in some cases may turn out to represent passenger rather than driver mutations. On the other hand, the localization within defined protein domains indicates functional relevance of most of the genetic alterations identified. Moreover, the mutations affect genes that are potentially relevant as target of pharmacologic intervention for these types of cancers. It is the case of the most promising alterations we found affecting the *EPHA3* and *EPHB2* genes in pancreatic adenocarcinoma and AVC samples, considering their emerging role as attractive therapeutic target in cancer [41].

## Supporting Information

Figure S1 Examples of somatic mutations identified in primary PDAC samples. The chromatograms refer to the sequence of tumor samples. A, homozygous mutation in BRAF (g.143148 G>T, p.G464V). B, heterozygous mutation in EPHB2 (g.152108 G>C, p.D283H). Arrows indicate the location of missense mutation. Numbers above the sequence traces are part of the software output. The nucleotide numbering uses the A of the ATG translation initiation start site as nucleotide +1, based on reference sequences provided in Supplementary Table S3.
(1.35 MB TIF)

Table S1 Clinical information on the pancreatic tumours included in the study.
(0.06 MB DOC)

Table S2 Pancreatic tumor cell lines included in this study.
(0.03 MB DOC)

Table S3 Protein kinase genes and primers used for PCR amplification and sequencing.
(0.24 MB DOC)
