# Supplementary figures and images for: Mutational Profiling of Kinases in Human Tumours of Pancreatic Origin Identifies Candidate Cancer Genes in Ductal and Ampulla of Vater Carcinomas
Source: PLoS One. 2010 Sep 8;5(9):e12653. doi: 10.1371/journal.pone.0012653 (PMC2935892; doi:10.1371/journal.pone.0012653)

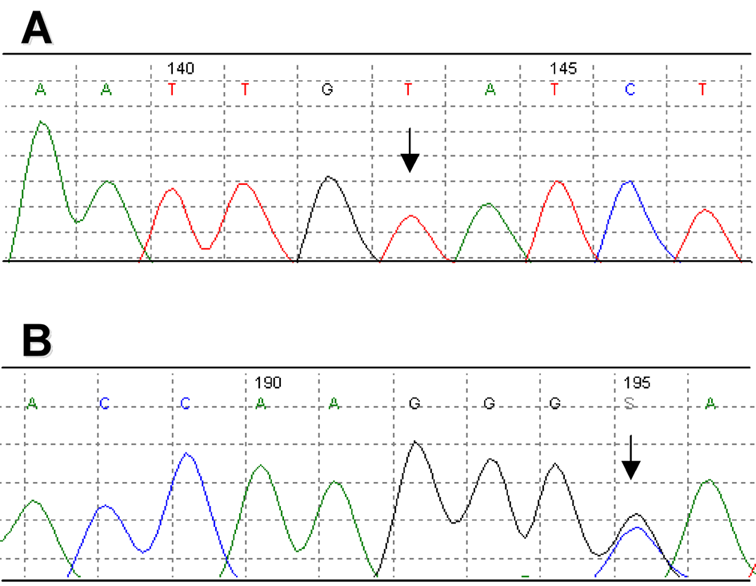

Supplement: Figure S1 — Examples of somatic mutations identified in primary PDAC samples. The chromatograms refer to the sequence of tumor samples. A, homozygous mutation in BRAF (g.143148 G>T, p.G464V). B, heterozygous mutation in EPHB2 (g.152108 G>C, p.D283H). Arrows indicate the location of missense mutation. Numbers above the sequence traces are part of the software output. The nucleotide numbering uses the A of the ATG translation initiation start site as nucleotide +1, based on reference sequences provided in Supplementary Table S3. (1.35 MB TIF) [file pone.0012653.s001.tif]
