# Supplementary material for: Mutational Profiling of Kinases in Human Tumours of Pancreatic Origin Identifies Candidate Cancer Genes in Ductal and Ampulla of Vater Carcinomas
Source: PLoS One. 2010 Sep 8;5(9):e12653. doi: 10.1371/journal.pone.0012653 (PMC2935892; doi:10.1371/journal.pone.0012653)
Supplement: Table S1 — Clinical information on the pancreatic tumours included in the study. (0.06 MB DOC) [file pone.0012653.s002.doc]

**Table S1.** Clinical information on the pancreatic tumours included in the study

| **Tumor Type** | **Sample ID** | **Age** | **Gender** | **Stage** | **Tumor Source** | **Tumor DNA source** | **Matched normal source** |
| --- | --- | --- | --- | --- | --- | --- | --- |
| Pancreatic ductal adenocarcinoma | 369 | 61 | Male | II | Primary Tumor | Xenograft | Pancreas |
|  | 370 | 52 | Female | III | Primary Tumor | Xenograft | EBV immortalized B lymphocytes |
|  | 371 | 57 | Male | II | Primary Tumor | Xenograft | Duodenum |
|  | 374 | 62 | Female | II | Primary Tumor | Xenograft | Pancreas |
|  | 377 | 52 | Male | III | Primary Tumor | Xenograft | Pancreas |
|  | 379 | 62 | Female | III | Primary Tumor | Xenograft | Spleen |
|  | 380 | 57 | Male | III | Primary Tumor | Xenograft | Duodenum |
|  | 382 | 44 | Female | II | Primary Tumor | Xenograft | Duodenum |
|  | 384 | 69 | Female | II | Primary Tumor | Xenograft | Spleen |
|  | 387 | 51 | Female | II | Primary Tumor | Xenograft | Pancreas |
|  | 546 | 70 | Female | III | Primary Tumor | Xenograft | Pancreas |
|  | 547 | 58 | Male | III | Primary Tumor | Xenograft | Pancreas |
|  | 548 | 54 | Male | III | Primary Tumor | Xenograft | Spleen |
|  | 549 | 49 | Male | III | Primary Tumor | Xenograft | Spleen |
|  | 550 | 69 | Male | II | Primary Tumor | Xenograft | Pancreas |
|  | 553 | 39 | Male | III | Primary Tumor | Xenograft | Duodenum |
|  | 554 | 66 | Female | II | Primary Tumor | Xenograft | Pancreas |
|  | PP109 | 63 | Female | III | Primary Tumor | Short-term culture | Pancreas |
|  | PP117 | 80 | Male | III | Primary Tumor | Short-term culture | Pancreas |
|  | PP147 | 60 | Female | III | Primary Tumor | Short-term culture | Pancreas |
|  | PP161 | 67 | Female | III | Primary Tumor | Short-term culture | Pancreas |
|  | PP244 | 68 | Male | II | Primary Tumor | Short-term culture | Pancreas |
|  | PP391 | 77 | Male | II | Primary Tumor | Short-term culture | Pancreas |
| Ampulla of Vater Cancer | 555 | 77 | Female | III | Primary Tumor | Primary Tumor | Duodenum |
|  | 557 | 64 | Male | IV | Primary  Tumor | Primary Tumor | Duodenum |
|  | 560 | 66 | Female | II | Primary Tumor | Primary Tumor | Pancreas |
|  | 562 | 69 | Male | III | Primary Tumor | Primary Tumor | Pancreas |
|  | 563 | 53 | Male | III | Primary Tumor | Primary Tumor | Pancreas |
|  | 100p | 23 | Female | II | Primary Tumor | Primary Tumor | Pancreas |
|  | 107p | 69 | Male | II | Primary Tumor | Primary Tumor | Pancreas |
|  | 119p | 68 | Female | III | Primary Tumor | Primary Tumor | Duodenum |
|  | 132p | 73 | Female | III | Primary Tumor | Primary Tumor | Pancreas |
|  | 135p | 72 | Male | II | Primary Tumor | Primary Tumor | Duodenum |
|  | 142p | 31 | Male | II | Primary Tumor | Primary Tumor | Pancreas |
|  | 143p | 62 | Female | IV | Primary Tumor | Primary Tumor | Duodenum |
|  | 145p | 55 | Male | IV | Primary Tumor | Primary Tumor | Pancreas |
|  | 160p | 75 | Female | II | Primary Tumor | Primary Tumor | Duodenum |
|  | 341p | 64 | Female | III | Primary Tumor | Primary Tumor | Pancreas |
|  | 564 | 44 | Female | II | Primary Tumor | Short-term culture | Pancreas |
