# Supplementary material for: Mutational Profiling of Kinases in Human Tumours of Pancreatic Origin Identifies Candidate Cancer Genes in Ductal and Ampulla of Vater Carcinomas
Source: PLoS One. 2010 Sep 8;5(9):e12653. doi: 10.1371/journal.pone.0012653 (PMC2935892; doi:10.1371/journal.pone.0012653)
Supplement: Table S2 — Pancreatic tumor cell lines included in this study. (0.03 MB DOC) [file pone.0012653.s003.doc]

**Table S2**. Pancreatic tumor cell lines included in this study.

| **Name** | **Origin** | **Source of tumor cells** | **ATCC/DSMZ**  **Number** | **Obtained from** |
| --- | --- | --- | --- | --- |
| AsPC-1 | Pancreas adenocarcinoma | Ascites | CRL-1682 | ATCC |
| BJ | n.a.^a^ | n.a.^a^ |  | Moore et al. 2001^b^ |
| Ger | Pancreas adenocarcinoma | Primary tumor |  | Moore et al. 2001^b^ |
| HPAF-II | Pancreas adenocarcinoma | Ascites | CRL-1997 | Moore et al. 2001^b^ |
| Mia-PaCa2 | Pancreas carcinoma | Primary tumor | CRL-1420 | ATCC |
| Paca3 | Prostate adenocarcinoma | Primary tumor | CRL-1435 | Moore et al. 2001^b^ |
| PaTu 8902 | Pancreas adenocarcinoma | Primary tumor | ACC-179 | Moore et al. 2001^b^ |
| PANC-1 | Pancreas epitheloid carcinoma | Primary tumor | CRL-1469 | ATCC |
| PC | n.a.^a^ | n.a.^a^ |  | Moore et al. 2001^b^ |
| PSN1 | Pancreas adenocarcinoma | Primary tumor |  | Moore et al. 2001^b^ |
| PT-45P1 | Pancreas carcinoma | Primary tumor |  | Moore et al. 2001^b^ |
| SKPC1 | Pancreas adenocarcinoma | Primary tumor |  | Moore et al. 2001^b^ |
| T3M4 | Pancreas adenocarcinoma | Lymph node metastasis |  | Moore et al. 2001^b^ |

NOTE: The name, origin, source, and supplier of the tumor cell lines are specified. ATCC: The American Type Culture Collection, Manassas (VA),

USA; DSMZ: German Collection of Microorganisms and Cell Cultures, Braunschweig, Germany.

^a^ n.a., not available.

^b^ Detailed information on suppliers in Moore et al., 2001 [26].
