# Supplementary material for: Mutational Profiling of Kinases in Human Tumours of Pancreatic Origin Identifies Candidate Cancer Genes in Ductal and Ampulla of Vater Carcinomas
Source: PLoS One. 2010 Sep 8;5(9):e12653. doi: 10.1371/journal.pone.0012653 (PMC2935892; doi:10.1371/journal.pone.0012653)
Supplement: Table S3 — Protein kinase genes and primers used for PCR amplification and sequencing. (0.24 MB DOC) [file pone.0012653.s004.doc]

**Table S3.** Protein kinase genes and primers used for PCR amplification and sequencing.

| **Gene Symbol** | **Gene ID** | Gene Name | **Exons** | **Forward primer** | **Reverse primer** | **Sequencing Primer** |
| --- | --- | --- | --- | --- | --- | --- |
|  | 208 | v-akt murine thymoma viral oncogene homolog 2 | 10 | GAGGGTGAGGCTGTGTGTG | GAGGATGGAGGAGAAATGAGG | GGAGGGTTGATGTCCAGGAG |
| AKT2 |  |  |  |  |  |  |
|  |  |  | 11 | GTGTGAGGCCAAGGGTAGG | TGTGGGGACGACACACTG | GTGTGTTTCCAGCCCAGATG |
|  |  |  | 11 | GGACACCAGGTCTTATTCCTTC | ATAAAGCCATCTGGCATCAA | CAAGAATCTTCCCAAATGTAATCA |
|  |  |  | 17 | ACAGATGTGAGCCACTGTGC | GAGGCCTCTTATACTGCCAAA | GCCCAGCCTGATTAGGTAAA |
|  |  |  | 21 | GCAAGGTGAGTATGTTGGCA | GATCAGCCTACGGGAAAAGA | GGCATATTCCACATAATGACAAA |
|  |  |  | 27 | CCTTGTTTGGCTGATTTTCATAC | AGACATTGAAGGTGTCAACCAA | AGTCTACAGGTTGGCTGCATAGA |
|  |  |  | 34 | ACGCCTGGCTAATTTTGTATTTT | TGTGTGAAGTATCATTCTCCATGA | CACTCGGCCTTAAGGTTAATTCT |
|  |  |  | 35 | TGAATGACTAGTGAAAGTCCTTTGA | CAATTTAACAGTCATGACCCACA | TTTTTCAGTGGAGGTTAACATTCA |
| ATM | 472 | ataxia telangiectasia mutated | 38 | ATTCCTTCAGAACCAATTTTGTG | ACACTTTGCAACACCTTCACCTA | AGCATAGTGGGAGACAGACACAT |
|  |  |  | 42 | TTCCATGTTTTCAGGATCTTCTC | GGAGTATCCCTGAATGTTTAGCC | GGCTGTGTAAATATCCACCAACA |
|  |  |  | 47 | CCCTGACAAGTAGTTAAGTCCTCAAT | GACCACATGATGGACTGATAGAA | GTCCTCAATGAATGGTAGTTGCT |
|  |  |  | 49 | TGGACAAGTTTGCAATAGTTCAT | CCGACCTTTAGAGCTCAAA | GACCAAGTCACTCTTTCTATGCAA |
|  |  |  | 50 | TTTGTCCTTTGATGCTTAGGAAG | TTATGTGTAGAGCACTGGACCAA | TTTCCCTGGGATAAAAACCC |
|  |  |  | 52 | CCTCTGCCTTTTTCTCACACAT | AGCCTTGAACCGATTTTAGATG | ACGCTCTACCCACTGCAGTATC |
|  |  |  | 54 | CTCAATCAGAGCCTGAACCAC | GAGGTGTAGAACAGAAGGCAATG | GGCCAGTGGTATCTGCTGAC |
|  |  |  | 58 | TTCCCTGTCCAGACTGTTAGC | TGATTTAATAATGAAGATGGGTTGG | TTCATCTTTATTGCCCCTATATCTG |
|  |  |  | 25 | CCCCTAAGAAGGATACTGGAAAA | TGTGTGCTAGGCATTCAGATAGA | GATTGCAGTAAAGGCAAGGC |
|  |  |  | 35 | GTGGCTACAGAGAGCCAACTG | CATGTGCTTTGCCATATAGACTT | TGAATGAATGAGATGAAGCAATG |
| ATR | 545 | ataxia telangiectasia and Rad3 related | 40 | CACAGACTGCTGAACCTTTGTAA | AAACGTTACGGTGAATGTGAATC | TTGCCATCAGTACAAATGAGTTTAG |
|  |  |  | 43 | TGAAAAATGTTCCTTTATTCTTG | CATTGACATTACTGCAGTGGAAC | TTGAATAAAAGTAGATGTTTCTTGTCC |
|  |  |  | 45 | GTAAAAGCAAAGGCAGAGCTACA | CAAACATATGTAGGGGCCAATAA | GCTACATGGCTCCTTCATGTTAC |
|  |  |  | 3 | GAACTACTGATAGGGCTGGGTCT | ACCCAGGGTAGGAGGTAGTGTAA | GGAAGGATACAATGAAAGAGGAAG |
| AURKC | 6795 | aurora kinase C | 5 | CTGAGTGGCTCAATGTAGGTTCT | AAACTGGGTCATTCCTAACTGGT | AAAGGAGGAAATGGAGTTACTGG |
|  |  |  | 6 | AGCATGAGCTAAATCACTGTGTG | AGACAACCCTGTGTGTGTGTG | TAGGGTCTCCACATCTCAATGAA |
|  | 673 | v-raf murine sarcoma viral oncogene homolog B1 | 11 | TTAAGGGGATCTCTTCCTGTATC | CAAAATAGTTTATTGATGCGAACAG | TGTATCCCTCTCAGGCATAAGG |
| BRAF |  |  |  |  |  |  |
|  |  |  | 15 | AGCAGGTTATATAGGCTAAATAGAAC | TGATTTTTGTGAATACTGGGAAC | TCTTACCTAAACTCTTCATAATGCTTG |
|  | 6046 | bromodomain containing 2 | 3 | GCACCTGGATTCATCAGACTATT | AGCATCTACACTAGGCAGACCAC | ATTCTTGCTCATCCCACACC |
| BRD2 |  |  |  |  |  |  |
|  |  |  | 11 | ACAGTGGGAACATACTGGAAGAG | TAAGGTTCCCAAGTTCCCTTCT | CAAACAGACCCCACCATCTT |
|  | 780 | discoidin domain receptor tyrosine kinase 1 | 13 | GAGCTTCACTTTCTCTGCCTGTA | AAGAGAATGGGAAAGGGATACAA | GCCTGTAAGATGGTGCTGATAGT |
| DDR1 |  |  |  |  |  |  |
|  |  |  | 19 | ACCAGAGCATGGAGAGGAAAG | ACAGGTACACCTGCATTGTGG | CAAGGGAGAGGAGTTGGAAA |
|  |  |  | 3_1 | GGAACCCTGAATATGTACAGCAA | GCTTCTTAGCATTTAGACCCAAG | ATCACATGTTTTGTGGCCTTTAC |
|  |  |  | 3_2 | CCCCATGACACCTGAACAAG | TCATGCTCAGCAGCTCAAAC | ACTCACAGCCTTCGAACACC |
| DYRK2 | 8445 | dual-specificity tyrosine-(Y)-phosphorylation regulated kinase 2 | 3_3 | TCCTCAAGGTCATTGGGAAG | ACGTGTAGACACGCTGATGC | GGCCTACGATCACAAAGTCC |
|  |  |  | 3_4 | AGAATTTCACCTTCCGCAAC | GCATGCCCAACAGTTCAATC | TTGAGCTGCTGAGCATGAAC |
|  |  |  | 3_5 | TTAAAGCAGCAGGGTAGAAGC | GGATCATCACACCCCTTCAG | GTTACGAGCATCAGCGTGTC |
|  |  |  | 3_6 | TTTGTGAGCTCCAAGGGTTATC | GTAAGGCTCAGCAATGTCGTATC | GTTACTGCACTGTCACGACTCTC |
|  |  |  | 15 | AACAGTGACCATATCAAGCAGGT | CTCAAATACAAACCTCGGCAAT | GGTGCAATCACAGAATAACTGGT |
|  |  |  | 18 | CAGAGCCTGTGTTTCTACCAACT | GGAAATATACAGCTTGCAAGGAC | AAGCTCTGTAGAGAAGGCGTACA |
|  |  |  | 19 | GGTAACATCCACCCAGATCACT | CTGTCTCTAAGGGGAGGGAGTTA | CCACTAGAGCTAGAAAGGGAAAGA |
| EGFR | 1956 | epidermal growth factor receptor | 20 | GTAAACGTCCCTGTGCTAGGTC | CATGGCAAACTCTTGCTATCC | CATTCATGCGTCTTCACCTG |
|  |  | (erythroblastic leukemia viral (v-erb-b) oncogene homolog, avian) | 21 | GAGGAACTGGATGGAGAAAAGTT | TCAGGAAAATGCTGGCTGAC | CCTTTCCATTCTTTGGATCAGTAG |
|  |  |  | 22 | AGACTGAAATCCCCTGTTGC | TTCCTCTGCCGTGTTTTCTC | ATCTGGCTCGTCTGTGTGTG |
|  |  |  | 26 | CCTTCACAATATACCCTCCATGA | CCATGTAAAATAGAGCCATAGTGGA | ATTCAGGAAAAGTGGATGAGATG |
|  |  |  | 3_1 | GTCCATTAACTCAGAGAACCTTGC | TCTTTGTAAACTGATGCTCTCG | GGCTCTGACACCCTTATGTTG |
|  |  |  | 3_2 | CCAGGAACTCAGCTCAGAAGA | GAGTCCATGGGTACCGTGTC | TCCATTGGTTTTAGGAACTTGC |
|  |  |  | 3_3 | TAGGTCCTGTCAACAAGAAGGG | TGCCAAGTGAGTTGAGACTGAG | GATGTTGGTGCTTGTGTTGC |
| EPHA3 | 2042 | EPH receptor A3 | 6 | GGGGCCAGATTAATATTCAAGAG | GAAACAGCTTCTTGCCTACACAG | GATAGACTCCATTTGCATGTTCC |
|  |  |  | 7 | GCCTAAGTTGCGTTCAGAGAATA | TCAGACATCTTTTGCAAGATCC | AGAATCAGGTTTGAGTGGTTCAC |
|  |  |  | 13 | TTCAGCCTTGTATCCATTTGC | CGTTTTGGGTCATGATTTCTACT | CATATCTTTGTCTTGAGTGCTTAGG |
|  |  |  | 14 | GCTACTTCTGCTTGGTATTGAAA | ATGTTCCACTTCCTTGCACA | GAAATTCTGTCCGGTTTCAGA |
|  |  |  | 5 | CAGACCAGGAATTGGTGTGATA | TGAGGGAAAATTCTCACACAGAT | CACACAGATTTCAGTCAACTTGG |
|  |  |  | 6 | GCTACGTTCTCAAAATGCCAATA | GCAGCAGAACATGAATAGAGAGC | CCCTGTACACTTCTAGCCTCCAT |
| EPHA5 | 2044 | EPH receptor A5 | 8 | AGGGGAAATTTCAGCTAAGTGTG | GCTGATATATTGCTTGCCTGATT | GTCAGATCAAGGTGAGGAAGAGA |
|  |  |  | 15 | CTAGATCTTTTCAGTGAGGCAACA | CAAAACAGATCAGAGGGTAAGCA | TCAGTGAGGCAACACTGTCC |
|  |  |  | 18 | TGGAGGTATAATTTTAGAGCCTAACC | GATTACAAATTCTGTGGCTGAGG | GCCAACATGAAATATTGAGGTGT |
|  |  |  | 3_1 | TGCTGTTCCATTGATACTTTGAG | CGAATTTCAGTGTTGAGTTTGAG | CTTTGAGTAGCTTCATTTGTATCCTT |
|  |  |  | 3_2 | AGATGAGTCCCACGGAATTAAA | GCAAGAACCCTCAATTTCTTCAT | GCCAAACCAGTATACAAAGATCG |
| EPHB6 | 2051 | EPH receptor B6 | 3_3 | GATTCCTCCTCTTTGGTTGAAGT | CGACTTTACTGAAAGCACTTTGG | TTTCATACACCTTTCCCAGGAG |
|  |  |  | 14 | GGTTGGTTTCCTTCTGTCCTTT | AGGAAAGGGCATATAAACTTACAG | TCACCAAAGTTGATGCTTATCTTC |
|  |  |  | 4 | AGAGATGAGATTTTCCAGCAGTG | ACCAGGGAAAGACTGGCTGTAAC | TCTGAGCTTTTCTGCAGGTAGAC |
| EPHB2 | 2048 | EPH receptor B2 | 11 | GTTCTGTGTCTGCAAGGATGAGT | GAAGCTATGTCCATCGTTCCTTT | AGTGGACATGACAGGGAACAG |
|  |  |  | 12 | GTTCTCACCACCACTCTGAAGTT | TCTTCTCTGGCTCTGTGACTCC | CATTATGAGGATGATGCAGAGC |
|  |  |  | 18 | AGCAGAAGATCCGGAAGTACAC | CTCCCTTCTCCGCTGTAACTG | GAGACTGCTGCAGGAAACG |
|  |  |  | 19 | CTAGGGTGGTGAAGGATGTTTG | GGGTCCTTCCTGTCCTCCTA | GGATGTTTGGAGGACAAGTAATG |
| ERBB2 | 2064 | v-erb-b2 erythroblastic leukemia viral oncogene homolog 2, | 20 | AGGCTGGTACTTTGAGCCTTC | CAAAGAGCCCAGGTGCATAC | CTGTGGTTTGTGATGGTTGG |
|  |  | neuro/glioblastoma derived oncogene homolog (avian) | 21 | GTGTATGCAGATTGCCAAGGTAT | GCTCCTTGGTCCTTCACCTAA | GTATGCACCTGGGCTCTTTG |
|  |  |  | 22 | GCTGGGTGGAGTGGTGTCTA | AGCTCTCATCCTCCCTCCAG | TAGCCCATGGGAGAACTCTG |
|  |  |  | 23 | GCTACCTGCCATGATGCTAGA | AGGACCTCCCACCCTCCT | GCAGAACCTCTGGCTCAGTA |
|  | 2066 |  | 8 | TGTGGAGCAGTAACCAAGCA | AAACCTTGTTATATAGGCCCAGTTC | CACACATTGCATTTGACTGG |
| ERBB4 |  | v-erb-a erythroblastic leukemia viral oncogene homolog 4 (avian) |  |  |  |  |
|  |  |  | 25 | CCGGTGGTTGTGCTAAAGAC | GCACATCTTTTGTGGGTATGG | GGCATCACATTGATTTGAGCTA |
|  |  |  | 4 | TCATCACTAAGGGAGCAGTGG | GGGCAGTAAGATAGGAAACAGTG | GTTCATCTGGAACTGCACTAGC |
|  |  |  | 7 | AGAAGTGCTGGGAGGTTTACAA | GTAGACTGGCCCACGAAGACT | GAGGTTTACAACCCATCACTGG |
| FGFR1 | 2260 | fibroblast growth factor receptor 1 | 10 | CTCTGCCATTGTTGGGAAG | CCACTAGAATAGCAAGCAAGGAA | CCTGACTAAGAATGGGAAGGAGT |
|  |  |  | 12 | GAGAATCAAGTCCCAGGGAAA | CCAGATCCCGAGATAACACATT | GCCAAAGCAGCCTCTCTTAAC |
|  |  |  | 13 | GGTAGTAGAATGGATTTCCCAGGT | GTTCCCACCCTGGCATTAC | AGGCAGGAGATGGGAGGTT |
| FGFR2 | 2263 | fibroblast growth factor receptor 2 |  |  |  |  |
|  |  |  | 5 | AGCGAAATGATCTTACCTGTTTG | AAGAAATGTGATGTTCTGAAAGC | ACCTGTTTGGAGTTTACTCATGG |
|  |  |  | 7 | GCCACAGTGTTATTTCAAAGGTG | CCAGTTGTGGGTACCTTTAGATTC | TGACAGCCCTCTGGACAAC |
|  |  |  | 13 | TTGCTGAATTGCCCAAGG | TTCCAGGTTGTACAAGACATGC | CTAGCAAATGAGCATGTCCAAA |
|  |  |  |  |  |  |  |
|  |  |  |  |  |  |  |
|  |  |  |  |  |  |  |
|  |  |  | 5 | GAGCGGATGGACAAGAAGC | ACTGTACACCTTGCAGTGGAACT | CCATCTCCTGGCTGAAGAAC |
|  |  |  | 6 | GAGAACAAGTTTGGCAGCATC | CCTAGACCCAAATCCTCACG | GTACACGCTGGACGTGCT |
|  |  |  | 7 | CTCACCTGGGACAGAGGACT | GGCCGTAAGTCACAGGATTC | GGCTGAGGAGTTGGTGGT |
| FGFR3 | 2261 | fibroblast growth factor receptor 3 | 8 | TCACTGGCGTTACTGACTGC | CTCTACATGGTGAGCAGAGACG | CTGCAGAGAGGGCTCACAC |
|  |  |  | 9 | CATGTAGAGCCTAGGGTACTTTGG | TCTGACTGGTGGCTGTTTCA | TCATTCAATGCTGGTGGAAGT |
|  |  |  | 13 | GAGTACTTGGCCTCCCAGAA | TGAGTGTAGACTCGGTCAAACAA | AGGTGTGGGTGGAGTAGGC |
|  |  |  | 17 | GACCTGGACCGTGTCCTTAC | AAGCTCTGTGTAGCTGTCTCTCC | CCAGAGTGCTGAGGTGTGG |
|  | 2264 | fibroblast growth factor receptor 4 | 13 | CAAGAACATCATCAACCTGCTT | AGTGGAGCTGGAGAGACTGAGAG | GTTAGGGTGCAGAGCCAAAG |
| FGFR4 |  |  |  |  |  |  |
|  |  |  | 16 | AGGGCTCCTTCAGATTTGGT | GAGGAGGAGGACTGGAAAGTG | CTGTGGTGGGTCATGTCTGT |
|  | 2321 | fms-related tyrosine kinase 1 | 9 | CTGATGATCAAAGGTTTGAAATTG | GATGTTGTTACGCTGATTTTTGA | TGAGCTCAAAAACTTCAATGACC |
| FLT1 |  |  |  |  |  |  |
|  |  | (vascular endothelial growth factor/vascular permeability factor receptor) | 16 | TGTATACTGCCTGGCTTAGAAGG | GAAAGAAAGAGGGTCCAACATTT | GGGTTCGCTATGATAAACCATTT |
|  |  |  | 13_14 | ATTACTGAAACAGGATGTGAGAGA | CCCATTTGAGATCATATTCATATTC | TGTGAGAGATTATAATGAGTTGTCCAC |
|  |  |  | 14_15 | CTGCAGAACTGCCTATTCCTAAC | AAAGGATGGAAAAGAGAAGAAGG | AAAGCCAGCTACAGATGGTACAG |
| FLT3 | 2322 | fms-related tyrosine kinase 3 | 16 | ATGCCTGGCTTCTCTCATAATTT | AATTCCACTTGGGTTTGAGAGTT | TAATGCAGATTGACTCTGAGCTG |
|  |  |  | 20 | GCACAGCCCAGTAAAGATAAGAG | ACCATAAATCAAAAATGCACCAC | CACCGGTACCTCCTACTGAAGTT |
|  | 2475 |  | 2 | GCCGATAGCCCACAATTTAA | GATGGTACATTTAGCCCACACA | ACACTGCCTCTTTTAAACATCAGTC |
| FRAP1 |  | FK506 binding protein 12-rapamycin associated protein 1 |  |  |  |  |
|  |  |  | 55 | TTTCCCCTTTAGGGTAGGTAGG | TGGAACCTTTTCTGCTCAAAG | GGCAGGCGTTAAAGGAATAG |
|  |  |  | 1 | CCTCCGCGCTCTAGAGTTT | ATTCCGAGTTAGATCTGGCTTTC | CTCCCACCCTGCACTGAG |
| KDR | 3791 | kinase insert domain receptor (a type III receptor tyrosine kinase) | 17 | AAGGGAAACTAGAAGCAGGGTTA | CATTCTAATGGAGGAAGAGATGG | GCAGGGTTATAATAGGACCACTC |
|  |  |  | 24 | TGAACCCAGTGCTTGGTTAAGT | TGCACATCCTCATCACCTATGTA | TAGAGAGCTTCAGGACCTGTGTT |
|  |  |  | 2 | ATAAATAGCAGGGCAGCTTTGTC | GGCTCAGTCATCCATATGTCATC | CAGAAGATGGAACTCAGTATTGGA |
|  |  |  | 8 | AACTTGCTCCCTCAGGCTACT | TTCAAGTGAATTGCAGTCCTTC | GGGATTAGAGAGGGAGTGAAGTG |
|  |  |  | 9 | CTCACTAGGTCACCAAAGTGCTTA | TGGTAGACAGAGCCTAAACATCC | AGTATGCCACATCCCAAGTGTT |
|  |  |  | 10_11 | TAACCAAGGTGAAGCTCTGAGAC | AAGCCACTGGAGTTCCTTAAAGT | GTCAGTTTGGGACTGAGTGG |
| KIT | 3815 | v-kit Hardy-Zuckerman 4 feline sarcoma viral oncogene homolog | 12_13 | ACAAATGGTCCTTCAATTCCAC | AGCAAGAGAGAACAACAGTCTGG | TCAATTCCACCACCAGCAC |
|  |  |  | 14 | TTGGGACTAAGTAGTCTGATCCA | ACCTCAGAGTACCTCAGTTCATTT | CATGACCACCCTTGGGTATTT |
|  |  |  | 15 | AGACGGGAAATTTCTAACCTGAG | CTGCTACCATAAAGCAGAACTGG | GGTCCAGTCTATTATGTAGCAAAGG |
|  |  |  | 17 | GTGAACATCATTCAAGGCGTACT | GTAATGTTCAGCATACCATGCAA | AATGTGTGATATCCCTAGACAGGA |
|  |  |  | 18 | AGGTGATTGGGATCATCTGAGT | GGCTCTTACATTTCAGCAGGT | TCAGCAACAGCAGCATCTATAAG |
|  |  |  | 4 | ACTGCCTAAGAACTTTGTTGCAT | ATAGAATCGAATCCTGCCATCTT | ATCCTGCCATCTTCATTTGTC |
| MAP2K4 | 6416 | mitogen-activated protein kinase kinase 4 | 8 | GACGCTAGACATGGATTCCTCTA | CCATTCTTAGTGCAGTTGCAGA | CTGTCTACCCAGCTGTTGCTT |
|  |  |  | 9 | CCCAAGCTAACCTGTGTTTAATTC | TACCAATGCTGCTAAGACCAAGT | CCTGTGTTTAATTCAAGGCTTTAC |
|  |  |  | 14 | GATTGATTGCTGGTGTTGTCTC | AATGTCACAACCCACTGAGGTAT | TGCTGGTGTTGTCTCAATATCAA |
|  |  |  | 16 | TGAAGCTCATAAAGGGTTTGATA | AAAACAAATTTTCAGGATTAGGC | CCATAATTTCAGTGGTAGCTGAT |
|  |  |  | 17 | ACAAGATGCTAACTGTGTGGTTT | TTAAATGTGCATCTTTGGCTACT | TTACCATTTCATTGCTCTTCCTA |
| MET | 4233 | met proto-oncogene (hepatocyte growth factor receptor) | 18 | CTTGAGCCATTAAGACCAAACTA | TTTGCATAAGAAGAGAAAACAGC | GCTTAACTAGCATTGAACAGTGG |
|  |  |  | 19 | TCCTTCAGAAGTTATGGATTTCA | TATGAAGAAAACTGGAATTGGTG | TTCAAATACTGAAGCCACTTGTT |
|  |  |  | 20 | AGCCAAGTTTAGTTACCAAGACC | CCAGCATTTTAGCATTACTTCAT | CCAAAAAGAAAGACATGCTGTAA |
|  |  |  | 5 | AGTGAAAGAGAGAGAGATCTGGATG | GACTGAGAGACTGAAACTTGAGAAA | GAGAGAGATCTGGATGTAAGTAAAGC |
| NTRK2 | 4915 | neurotrophic tyrosine kinase, receptor, type 2 | 17 | TCTCCTCTTCATGCTAAGTCAGG | GTTATGACAGCCTCAGCAAACA | GCTAAGTCAGGCAGCATCTTT |
|  |  |  | 18 | TCTGTCTCTGTTGCTTGAGACTG | AATGGATGCCTCTGGGATCT | TGCTTGAGACTGTGAAGAAGTCA |
|  |  |  | 15 | AAGGAAGGAGTCAGGCCTTATAG | TTTCTCATCCTGAGAGGAAAGTG | GCCAGTTTCTTTCCAGGAGTAG |
| NTRK3 | 4916 | neurotrophic tyrosine kinase, receptor, type 3 | 16 | AATAAGCCATAGGCAGGATGG | AGAGTGACAGGGTTAATGGACAA | GCAGGATGGAAAGGGAAAT |
|  |  |  | 17 | TATCGTAGGTCTCCAAAGTCAGC | TCTGGGCTGAGATAGCTCTTATG | CATCAAGAGTGCATCTATGTGTGA |
|  | 10298 | p21 protein (Cdc42/Rac)-activated kinase 4 | 4_1 | CTCCTCTGTCCCCACCTTC | GCTCATGGGATACTCGCTGT | CTCCTGCTTAGGGAGCAGA |
| PAK4 |  |  |  |  |  |  |
|  |  |  | 4_2 | ACAGCGAGTATCCCATGAGC | CTGGGGAGGGAGAAGAGTG | AGGAGTGCAGGGAGAGGAG |
|  |  |  | 12 | GTGAACGTTGTTGGACTCTACTG | GTAAAGTTGTGTGCAAGGGAAA | GTCCAGTCACTGTGCTGCTT |
|  |  |  | 15 | TATGGTCTGCAGGACAATTCAT | GGTGGTTTGACTCTAAGTCTTGC | GCACTGAATCTGCAGACATGATA |
| PDGFRA | 5156 | platelet-derived growth factor receptor, alpha polypeptide | 17 | TCTGACCTCAGGCAATCCA | ACACTCCACTCACTGAAATCTGG | GCCTCTGCAACCTGATGATT |
|  |  |  | 18 | AGAGCTTTCTCTCTGTTGGGAGT | CACCGAATCTCTAGAAGCAACAC | GAGAAGGCCAGCCCTTTATATC |
|  |  |  | 19 | GCACAAGTTATTAAGAGCCCAAG | ATGGGCCTATCTCAGACACAATA | GGCCTCACACCAGGTTATCTTA |
|  |  |  | 23 | GGCAGAATCTTGCCATACTGT | TAAACATCAACCAAGTCCTCCTC | AAGGCTTTCGTTTGTCTCTGG |
| PDPK1 | 5170 | 3-phosphoinositide dependent protein kinase-1 | 10 | CCTGACCTCAGGTGATCTGC | CGGCAGCAAAGACTACAACC | GGCTACCAGGTTTGGGTTTC |
|  |  |  | 14 | GTCATCAGCCTGTGTAGTTGCT | TAAAAATGCTGCAAGGTTTCC | AATAACCGTCACACCCACGT |
|  |  |  | 2_1 | GTTTCTGCTTTGGGACAACCAT | CTGCTTCTTGAGTAACACTTACG | GATTCATCTTGAAGAAGTTGATGG |
|  |  |  | 2_2 | CTCCACGACCATCATCAGG | GATTACGAAGGTATTGGTTTAGACAG | ACTTGATGCCCCCAAGAATC |
|  |  |  | 3 | TCATCAAAAATTTGTTTTAACCTAGC | TATAAGCAGTCCCTGCCTTC | TCTACAGAGTTCCCTGTTTGC |
|  |  |  | 5_1 | TCTTGTGCTTCAACGTAAATCC | CGGAGATTTGGATGTTCTCC | AAAATAATAAGCATCAGCATTTGAC |
|  |  |  | 5_2 | TCTCAACTGCCAATGGACTG | CGGAGATTTGGATGTTCTCC | TTATTCCAGACGCATTTCCAC |
|  |  |  | 6 | TAGTGGATGAAGGCAGCAAC | TTTGTAGAAATGGGGTCTTGC | TCTGAACAAAAATTCCGTGGT |
|  |  |  | 7 | TGCCTTTTCCAATCAATCTC | AATTCCTGAAGCTCTCCCAAG | TTCCTGTTTTTCGTTTGGTTG |
| PI3KCA | 5290 | phosphoinositide-3-kinase, catalytic, alpha polypeptide | 8 | GGGGAAAAAGGAAAGAATGG | TGCTGAACCAGTCAAACTCC | TGAATTTTCCTTTTGGGGAAG |
|  |  |  | 10 | GATTGGTTCTTTCCTGTCTCTG | CCACAAATATCAATTTACAACCATTG | TTGCTTTTTCTGTAAATCATCTGTG |
|  |  |  | 13 | AAACTGACCCTGATTTGTTTTTTTG | AATATTCATCTGGACCTAGAAAATTTG | GAGAAGCTCATCACTGGTACAAAATAC |
|  |  |  | 14 | ACCTGAAACTCATGGTGGTTTTG | TGGCCTTATGAAGCAGGTATTATTT | CATTTTACAGACAAAGAAAATGAGTCC |
|  |  |  | 15 | GAGTGTTGCTGCTCTGTGTTG | TTGAGGGTAGGAGAATGAGAGAG | TCTCATGTGAGAAAGAGATTAGCAG |
|  |  |  | 19 | TCCTTATTCGTTGTCAGTGATTG | GTCAAAACAAATGGCACACG | TGCACCCTGTTTTCTTTTCTC |
|  |  |  | 21_1 | TGGGGTAAAGGGAATCAAAAG | CCTATGCAATCGGTCTTTGC | TGACATTTGAGCAAAGACCTG |
|  |  |  | 21_2 | TTGCATACATTCGAAAGACC | GGGGATTTTTGTTTTGTTTTG | TTTGTTTTGTTTTGTTTTTT |
|  |  |  | 11_1 | ATATGCTTGAAACCGTACTCACC | GCCCATATTCATTGCATAATGTT | TCACCAGTCAGCAATAACAAGC |
|  |  |  | 11_2 | AGGAAGATGATGGCCAAGATAG | TAACTGGCACTGAGTCATCTGAG | CCAAGATAGCTCTCCAAAGTGG |
| RPS6KC1 | 26750 | ribosomal protein S6 kinase, 52kDa, polypeptide 1 | 11_3 | ACAGTAAGGATAGCGCAAGTGAA | TTAGGATCTGAGCTGGGTAACAA | TGGAGACAGTGCTTCTAGGAGTT |
|  |  |  | 11_4 | GAGAAACACTATGCACAGGAGGA | CTCAGTTAATTGGAAGCAACAGC | GAGGATCCCAGGATGTTATTTGT |
|  |  |  | 14 | GCGGCACTGTGTTGATTACTAC | GAGTTGTGGGCTCTGCTAATCT | TCTGTACTTGCTGTCCATAAATCC |
|  |  |  | 1 | AGAACAATCGTTTCTGTTGGAAG | AGAGAAGGAAGGAAGACAGAACC | AATTTTGGAGAAGGGAAGTCG |
|  |  |  | 4 | TTCAGAGGGGTGGCTGAG | AATATCAGGACAAGCAGTGTGG | TCCAGAGCCCCTTTTCTG |
|  |  |  | 5 | CCTGCTGTTCCAGCAAGACT | CCAGATGTCCACCTTGAAGC | GTGTGCCTGGACTTCTGTGAC |
| STK11 | 6794 | serine/threonine kinase 11 | 6 | TGTGCACAAGGACATCAAGC | CCATCTGCCGTATGAGTTACATT | CTGTGGCCAGAGAGGGTCT |
|  |  |  | 7 | AGACAGAGGTGTCCTTGAGTCC | CTCTCCACTCAGTCCTCTCAATG | TCTGTCCCTGGGGTAGAGC |
|  |  |  | 9 | GACATCTGTCAGGCTTGGAGT | CCACACCTTTCAGCCATGT | AGAAGCTGTCCTTGTTGCAGA |
|  |  |  | 4_1 | GCATGAACCCACTTCCTGAC | CATGCTTCAGATTGATGTCTGAG | TTCCTGACAGTACTTACCTACCACA |
| TGFBR2 | 7048 | transforming growth factor, beta receptor II (70/80kDa) | 4_2 | AGCAGAACACTTCAGAGCAGTTT | TAAGAGCCAGGAGATATGGATCA | TCAAGATCTTTCCCTATGAGGAG |
|  |  |  | 7 | GCACTCAGTCAGCACATGTTAAA | TCCTGCTGCCTCTGTTCTTT | TCACTATAGCAACAAGGTCAGCA |
